# Virtual Scenarios of Earthquake Early Warning to Disaster Management in Smart Cities Based on Auxiliary Classifier Generative Adversarial Networks

**DOI:** 10.3390/s23229209

**Published:** 2023-11-16

**Authors:** Jae-Kwang Ahn, Byeonghak Kim, Bonhwa Ku, Eui-Hong Hwang

**Affiliations:** 1 Earthquake and Volcano Technology Team, Korea Meteorological Administration, Seoul 07062, Republic of Korea; propjk@korea.kr; 2 Earthquake and Volcano Research Division, Korea Meteorological Administration, Seoul 07062, Republic of Korea; 3 School of Electrical Engineering, Korea University, Seoul 02841, Republic of Korea

**Keywords:** earthquake early warning, borehole-seismometer, seismic sensor, virtual seismic scenarios, Generative Adversarial Network

## Abstract

Effective response strategies to earthquake disasters are crucial for disaster management in smart cities. However, in regions where earthquakes do not occur frequently, model construction may be difficult due to a lack of training data. To address this issue, there is a need for technology that can generate earthquake scenarios for response training at any location. We proposed a model for generating earthquake scenarios using an auxiliary classifier Generative Adversarial Network (AC-GAN)-based data synthesis. The proposed ACGAN model generates various earthquake scenarios by incorporating an auxiliary classifier learning process into the discriminator of GAN. Our results at borehole sensors showed that the seismic data generated by the proposed model had similar characteristics to actual data. To further validate our results, we compared the generated IM (such as PGA, PGV, and SA) with Ground Motion Prediction Equations (GMPE). Furthermore, we evaluated the potential of using the generated scenarios for earthquake early warning training. The proposed model and algorithm have significant potential in advancing seismic analysis and detection management systems, and also contribute to disaster management.

## 1. Introduction

Advancements in digital conversion and storage technology for earthquake records have prompted an upsurge in the utilization of artificial intelligence (AI) for earthquake analysis. A range of AI-driven models demonstrates high performance in analytics within their trained parameters. However, accurately analyzing the non-stationary seismogram for earthquakes remains a challenge. Even for earthquakes with similar magnitudes and epicenters, there is a difference in the wave time series of each event [1]. Therefore, the AI-based model for earthquake analysis encounters limitations in perfectly identifying patterns in seismographic signals [2].

Recently, many researchers have swiftly developed AI models focused on the detection and analysis of seismic data. It’s recognized that AI methodologies are more efficient than traditional empirical analysis methods conducted by humans [3]. This field is rapidly advancing, with machine learning (ML) [4,5,6], Convolutional Neural Networks (CNNs) [7], multi-feature fusion CNN [8], Recurrent Neural Networks (RNNs) [9], and attentive models [10] being widely implemented. However, since deep learning models solve given tasks by finding latent features in large amounts of data, their effectiveness may be negligible in environments with insufficient data. Thus, the piecemeal application of these models to new, untrained environments for earthquake predictive analytics has inherent limitations [11,12].

Generative Adversarial Networks (GANs) that can generate data have been used as a solution to data shortages [13,14,15]. GANs were first introduced by Ian et al. [16] and have been widely used in image processing. In earthquake research, GAN has been applied and employed to augment the data of seismograms [17]. For instance, Zefeng Li et al. [18] trained to simulate the signal detection in earthquake early warning (EEW) by learning the characteristics of first-arrival P waves using GANs and Random Forests. This method, however, only focuses on generating the initial P waves. The SeismoGen [19] aims to generate artificial seismograms akin to earthquakes, yet questions remain about their similarity to reality seismic characteristics. Li et al. [20] attempted to enhance the precision of P-wave and S-wave generation using a conditional GAN. Additionally, Wu et al. [15] leveraged it to address the scarcity of recorded seismic waves in earthquake detection research based on MEMS data. As AI continues to advance, various attempts are underway to simulate seismic waves. However, it is crucial to consider the diverse characteristics inherent in the waveform when generating seismic wave.

The waveforms allow us to infer a multitude of characteristics associated with seismic activity processes. These waveforms provide critical information for estimating aspects such as the energy level of the source (i.e., amplitude, frequency), the hypocenter (i.e., phase arrival time, polarization), and geological effects (i.e., attenuation, spectral content) [21,22,23,24,25]. Recently, the application of physics-informed machine learning (PIML) has demonstrated significant advancements [26,27], especially in suppressing overfitting in dynamic systems through the use of dynamic equilibrium equations. However, generating a comprehensive time-window waveform that accurately encapsulates these complex characteristics continues to pose a challenge, considering the multifaceted factors involved.

In addressing the question of whether we can produce artificial waves by incorporating various physical factors inherent in seismograms, this study navigates through two primary limitations: (1) the complexity of the geological conditions that pose challenges in labeling [28], and (2) the existing imbalance in data distribution across different labeling groups [29]. Additionally, the effectiveness of current models is often limited to specific regions, as dictated by their training data, which presents challenges in areas with low seismicity [17,30]. This backdrop underscores the need for novel approaches in applying Generative Adversarial Networks (GANs) in seismology. Our study unfolds in distinct stages to address these challenges. Initially, we explore a method to generate artificial seismic waves using a minimal set of seismic features, focusing primarily on the source and the path. We aim to reproduce an artificial wave that mirrors the epicenter distance in terms of the arrival time of the Phase and the attenuation of amplitude. This approach is pivotal in developing earthquake scenarios that can significantly enhance disaster management strategies.

In this study, we pursued a method for generating artificial seismic waves based on a minimal set of seismic features, with the primary considerations being the source and the path. We tried to produce an artificial wave for the epicenter distance that mimics the arrival time of the Phase and the attenuation of amplitude. Our aims were to develop earthquake scenarios that could contribute to effective disaster management [31]. To achieve this, we focused on two main aspects: simulating waveforms that closely resemble actual earthquake events and verifying the accuracy of these generated scenarios. By adopting this two-pronged approach, our study contributes a novel perspective to the field of seismic analysis and presents potential advancements in both theoretical understanding and practical applications in disaster preparedness and response.

## 2. Selection of GAN

Generative Adversarial Networks (GANs) are a deep learning model composed of two opposing networks—a generator and a discriminator—that is used for unsupervised learning [16]. In the training process of GAN, the generator constantly generates fake data, and the discriminator learns to distinguish real data from fake data. By learning while competing with itself, the generative model generates more and more realistic data, and vice versa, allowing it to better distinguish between real and fake data. Ultimately, the generator aims to become a generative model that generates fake data similar to real data. The goal of GANs is to create a model that can generate new data that is similar to the real data distribution, which has applications in various fields, such as image synthesis and speech recognition. The equation used for this is referred to as a loss function and is shown in Equation (1).

(1) $$\underset{G}{\min}\underset{D}{\max}V\left(D,\;G\right)=E_{x\sim p_{data}(x)}\left[\log D(x)\right]+E_{z\sim p_{z}(z)}\left[\log\left(1-D(G\left(z\right))\right)\right]$$

where the symbol ‘E’ refers to the expected value, $x\sim p_{data}(x)$ is the distribution of real dataset x, and $z\sim p_{z}(z)$ is a randomly assumed probability distribution (e.g., uniform distribution, gaussian distribution). The generator $G\left(z\right)$ tries to deceive the discriminator D(x) by making $D(G\left(z\right))$ = 1 in order to minimize the loss value of the model. On the other hand, D aims to maximize the loss value of the model by making D(x) *=* 1 for actual data x and $D(G\left(z\right))$ = 0 for fake data generated by $G\left(z\right)$. Ideally, the GAN’s learning mechanism ends when the discrimination probability of the discriminator converges to 50%.

Although GANs have the ability to generate realistic samples, we cannot control which samples are created [32]. Conditional GAN(CGAN) allows you to control which data the GAN will generate. To consider seismic characteristics, CGAN can be used. CGAN that utilizes label information during the training process is expanded by incorporating the label information into both the generator and discriminator [33]. The CGAN- based algorithm is of similar characteristics to conventional ground-motion, where specific conditions (i.e., attenuation relationship for peak amplitude based on epicenter distance and site condition) are input into the model [34,35]. Because it is difficult to control sampling, CGAN simply generates random shapes of data from random samples that are considered only conditional input data.

**Figure 1:**
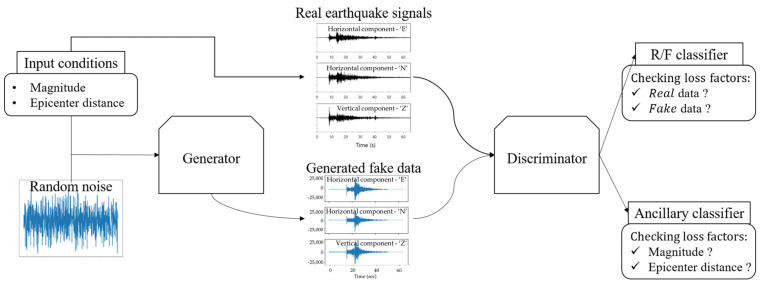
A schematic diagram illustrating AC-GAN models for synthesis earthquake.

Auxiliary classifier GAN (ACGAN) is a variant of CGAN that incorporates random noise and class label information, where the discriminator is composed of two classifiers that classify real/fake and class [36]. ACGAN improved the performance of CGAN by adding auxiliary classifier learning to discriminate data classes. The auxiliary classifier helps the model generate more realistic samples by determining which class the added condition belongs to in the generator [36]. To reflect the characteristics of magnitude and epicenter distance on seismic waveforms, we propose the virtual earthquake generation model based on ACGAN, as shown in Figure 1. Although conventional ACGAN models generally use discrete class labels as conditions, we used continuous information such as earthquake magnitude and epicenter distance as conditions in our proposed model. Therefore, we tried to develop the ACGAN to be able to simulate true earthquakes by accurately measuring an earthquake, which decreases as the recording point becomes further from the epicenter for the same-scale earthquake. We expected that the proposed model could be designed to simulate true earthquakes and achieve a more accurate representation of earthquake characteristics by an auxiliary classifier. The loss function of the proposed model is as follows:

(2) $$L_{D}=-E_{x\sim p_{data}(x)}\left[\log D(x|y)\right]-E_{z\sim p_{z}(z)}\left[\log\left(1-D(G\left(z|y\right))\right)\right]$$

(3) $$L_{G}=-E_{z\sim p_{z}(z)}\left[\log D(G\left(z|y\right))\right]-E_{z\sim p_{z}(z)}\left[\log P\left(C=c|G\left(z|y\right)\right)\right]$$

where the symbol ‘E’ refers to the expected value, $L_{D}$ and $L_{G}$ correspond to the loss function of the discriminator of the ACGAN model and the loss function of the generator, respectively. $L_{D}$ is identical to the loss function of the discriminator part except for the generator of the GAN. In contrast, $L_{G}$ refers to the loss function of the generator of ACGAN, and c (or ‘C’) in the Equation (3) is related to the ‘class’ (i.e., earthquake magnitude *M_L_*, and epicenter distance *R*). Thus, {$-E_{z\sim p_{z}(z)}\left[\log P\left(C=c|G\left(z|y\right)\right)\right]$} represents the probability that the fake sample belongs to a given class. $L_{G}$ is a new loss function in addition to the basic CGAN architecture.

## 3. Training Data

The digitally recorded earthquake data could be utilized for conducting earthquake hazard assessments based on statistical and probabilistic estimates. A prime example is Ground Motion Prediction Equations (GMPEs). GMPEs offering empirically simple solutions predict ground motion based on statistical and probabilistic methods [37,38]. Empirical models, like GMPE, depend on both the amount and quality of the data. Therefore, the models might exhibit varied optimized functions depending on factors such as event frequency, magnitude, and source location. Notably, diverse observation conditions also make it difficult to optimize normalized models.

This study considers the foundational theory of the GMPE. The GMPE identifies three primary effects: (1) source effects, which are related to the characteristics of the earthquake’s energy and origin (i.e., magnitude, focal mechanism, depth, and stress drop); (2) path effects, which concerns the seismic wave’s path from the earthquake source to the observation site (i.e., distance, attenuation, local geological structure); (3) site effects, which are linked to the unique conditions of the location where the ground motion is predicted (i.e., soil or shallow layer conditions, geological features, and topography). Of these, the attenuation of seismic waves is determined by the amount of energy released at the epicenter distance [39]. For site effects, amplification or de-amplification is contingent on local characteristics and could not be categorized as an attenuation function.

**Figure 2:**
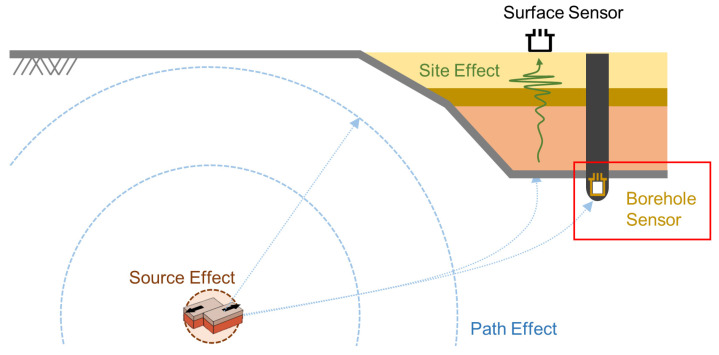
Schematic of seismic wave propagation and seismometer locations: the red box highlights the sensors utilized in ACGAN learning.

Based on these physical features, we proposed the concept illustrated in Figure 2. The seismic record is influenced by location characteristics such as geology, geometry, and depth [40,41,42,43]. Although surface sensors register all three effects, borehole sensors are largely unaffected by the site effect [43]. Therefore, we exclusively used borehole sensors to minimize variance due to site effect and focus solely on attenuation. These concepts have already been cited as important in research on the Korean Peninsula [44].

This study utilizes kik-net data (https://www.kyoshin.bosai.go.jp, accessed on 30 July 2020) and Korea Meteorological Administration (KMA) data (http://necis.kma.go.kr/, accessed on 31 March 2022) as its basic dataset. The seismic records used in this study were collected only at borehole sensors from 1997 to 2021. 

The data contains source information (i.e., Origin Time, Latitude, Longitude, Depth, and Magnitude) and Station information (i.e., Station Code, Station Latitude, Station Longitude, Station Height, Record Time, Sampling Frequency, Direction, and Scale Factor). Therefore, we were able to configure labeling when applying GAN. In this study, we grouped the data based on the important functions of local magnitude (*M_L_*) and Epicenter distance (*R*, unit is km). Table 1 summarized the amount of data applied in this study.

A total of 117,331 datasets were used for training, which were organized into seismic waveforms with two horizontal and one vertical component. However, the R and M could not be further refined due to the data imbalance. The lack of data for *M_L_* ≥ 5 and *R* < 40 km hindered the convergence of the loss function during ACGAN training.

## 4. Results and Validation

The developed program in this study can generate seismic waves. We verified this by generating a three-component (i.e., twice horizontal and vertical) seismogram based on the set magnitude and target separation distance. The generating data format is mini-SEED. However, our program can only generate less than 120 km from the epicenter due to the training data set.

**Figure 3:**
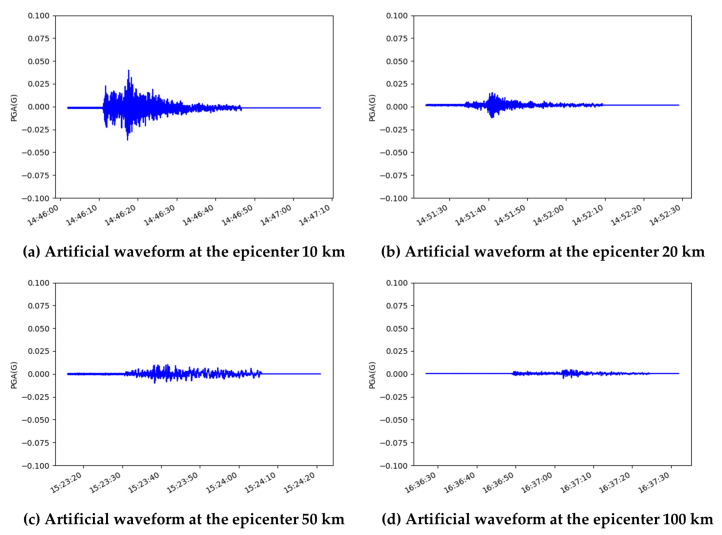
Generated artificial horizontal seismic waveforms using proposed GAN model.

Figure 3 illustrates the seismic waveforms generated by our model for four randomly chosen input noise signals, categorized into six different epicenter separation bins. The generated data exhibit a diverse range of patterns while adhering closely to the fundamental time series seismic wave shape. The lack of a regular pattern in the input data is an advantage, as it enables the model to generate a wide variety of data. Additionally, the difference in P-S arrival time is being simulated, which confirms the superiority of the seismogram generation. 

Additionally, we designed a program to generate artificial seismic waves based on the KMA borehole-type seismic network. The verification for the final version focused on the attenuation of seismic waves, which was based on actual earthquake locations. Table 2 proposes a total of 10 events used for the verification. Waveform comparisons were excluded, as a perfect simulation of seismic waves generated by virtualization is impossible, and time-varying acceleration characteristics may differ.

**Figure 4:**
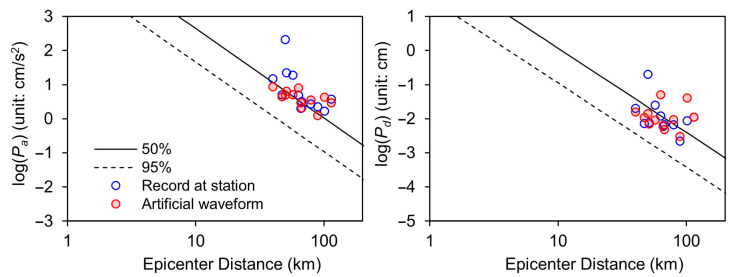
Comparison of vertical amplitude and attenuation relationship between record data and generated artificial waveforms.

**Figure 5:**
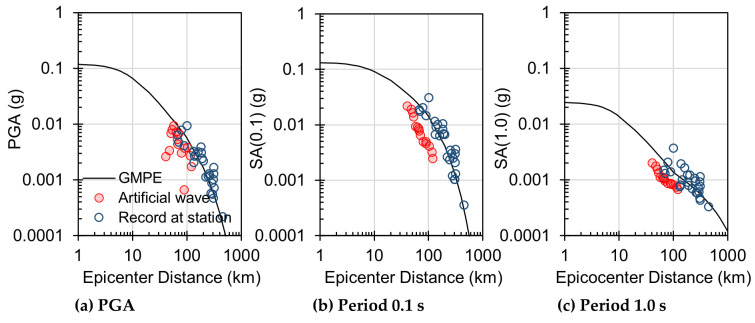
Comparison of attenuation relationship based on GMPE between record data and generated artificial waveforms.

Figure 4 presents the results for the Gyeongju earthquake that occurred on 12 September 2016. It specifically illustrates the simulated attenuation performance of the initial P-wave and juxtaposes this simulation with the regression curve for a depth of 20 m, as presented in the study by Cho et al. [45]. This comparison effectively demonstrates that the attenuation of peak acceleration (Pa) and peak displacement (Pd) is well captured by the simulation. In Figure 5, it focuses on the Pohang earthquake, which occurred on 15 November 2017. This figure undertakes a comparative analysis of the attenuation for maximum acceleration within rock parameters, specifically using Ground Motion Prediction Equations (GMPE) for bedrock as proposed by Jang et al. [44]. Notably, the amplitude attenuation displayed in Figure 5 exhibits characteristics that align closely with those predicted by the statistical estimation model. This similarity underscores the effectiveness of the GMPE model in replicating real-world seismic data characteristics, thereby reinforcing the potential of these models in seismic analysis. Figure 4 and Figure 5 collectively provide significant insights into the seismic attenuation characteristics for these two major earthquakes. They highlight the capability of current modeling techniques in replicating and predicting seismic wave behaviors, which is crucial for enhancing earthquake preparedness and response strategies.

The objective of this study, using ACGAN, was to create earthquake scenarios in areas of low seismicity. So, we verified possible the simulation of early warnings utilizing the proposed program. For our simulations, we used ElarmS 3.0 [46], which has been optimized in previous studies [45]. The produced waveforms were transitioned to tankfiles and tested with the simulation tool proposed by Lim et al. [47].

**Figure 6:**
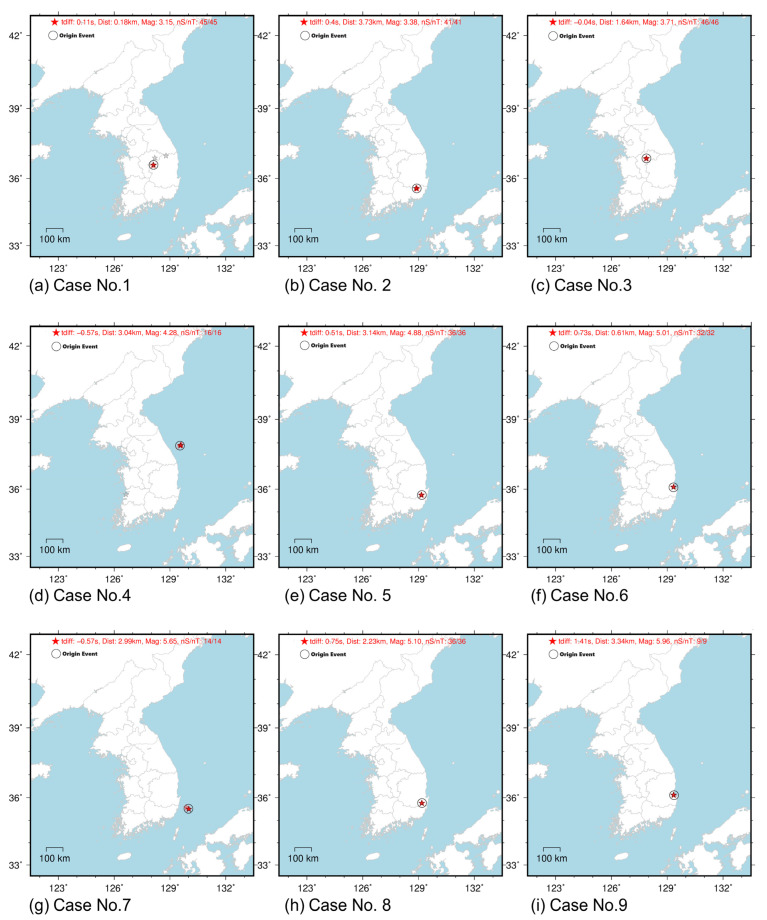
Comparative analysis of source information: Circles represent source information from real event records, calculated by Antelope. The red star indicates the results of the epicenter and magnitude form nS (number of stations) and nT (number of Triggering points). The gray star denotes the calculation of source information before reaching a specific convergence phase in the analysis.

Figure 6 shows the results of inputting the scenarios of Table 2 into ElarmS3.0 with a generated scenario wave set. The resulting errors in magnitude, location, and time of earthquake occurrence are summarized in Table 3. Figure 6 presents a comparative analysis of source information, specifically the epicenter, magnitude, and event origin time, using both the ElarmS and the Antelope systems (version 5). ElarmS utilizes only the initial P-wave to determine the source information, and the results of the GAN are inputted here. Antelope [48], on the other hand, uses the initial P-waves and S-waves from real event records to determine the source information, which tends to be close to the true values. This figure aims to showcase the effectiveness of generative waves. Thus, the comparison aids in understanding the reliability and potential limitations of the GAN system for earthquake early-warning event detection and analysis.

The magnitude error was found to be up to 0.67, which is an acceptable range of error given the range of magnitudes labeled during fine-grained training. The location error was up to 3.7 km, and the time error was up to 1.41 s. The error in the analysis with artificial seismic waves was not significant, and it was confirmed that the P-S time was well simulated. Therefore, it is confirmed that our model can be used for the verification of early earthquake warnings based on the attenuation characteristics of P-wave and maximum acceleration, and a good simulation of P-S arrival time.

## 5. Limitation

### 5.1. Data Limitations

This study was restricted to drilled stations and epicenters within 120 km, and only light and medium earthquakes (3.0 ≤ *M_L_* < 6.0) were considered, with amplitude attenuation trends confirmed. The site effect was not trained, and for estimating IM at a specific site surface, passive methods such as transfer functions or one-dimensional ground response analysis (Kwak et al. [49]) should be used.

### 5.2. Study Limitations

Manual preprocessing for seismic training and validation is complex and requires global, unified rules. The number of AI-generated seismic data for each seismic event is still limited, and more data for comparison/validation is needed for greater statistical reliability. The variance (regression error) of AI-generated earthquakes can be useful for handling outlier data, whereas a smaller variance is beneficial for generating data that better simulates amplitude decay. The optimized loss function of the GAN model can partially control this variance. Finally, GAN models are technically challenging to train in AI, requiring significant computational resources and expertise.

## 6. Conclusions

In conclusion, this study has successfully utilized AC-GAN, an advanced artificial intelligence technology, to generate virtual earthquake data for the Korean Peninsula. The model used was an improved version of GAN that accurately reflects the characteristics of real seismic waves and was trained with a large amount of seismic observation records. The generated earthquake data was undistorted and responded appropriately to changes in input samples and epicenter distance. By inputting the virtual earthquake data into the ElarmS earthquake early warning system, the time, location, and magnitude of the earthquake source were simulated with high accuracy. This technology can be utilized for virtual earthquake simulation exercises specific to the Korean Peninsula and can supplement actual earthquake history by creating scenarios of earthquakes that can potentially occur in the region. This study could use important technology for earthquake preparedness and risk management in the Korean Peninsula.

Given the specific nature of the data used to train our GAN models, their applicability may not extend to all scenarios. Therefore, it is essential to integrate them with traditional interpretation methods for predicting seismicity that reaches the surface. Through refining and effectively combining these learning methods, we can enhance our ability to predict the impact of various earthquakes. Additionally, although the characteristics of the earthquakes we have analyzed primarily focus on the P-S phase (i.e., IM and Amplitude of wave), it is crucial to also consider a broader range of factors, such as stress drop, wave tails, and the clustering features of seismic waves. Developing a GAN model that can accurately simulate these aspects will further advance our earthquake analysis capabilities.

## Figures and Tables

**Table 1:** Waveform set used for training.

Magnitude	*R* < 20	20 ≤ *R* < 40	40 ≤ *R* < 60	60 ≤ *R* < 80	80 ≤ *R* < 100	100 ≤ *R* < 120	Total
3 ≤ *M_L_* < 4	6360	12,868	13,248	9718	3764	2534	48,492
4 ≤ *M_L_* < 5	2473	6856	11,096	13,815	11,320	10,918	56,478
5 ≤ *M_L_* < 6	336	899	1605	2509	3261	3751	12,361
Total	9169	20,623	25,949	26,042	18,345	17,203	117,331

**Table 2:** Validation scenario for earthquake cases.

Case No.	Event Region in South Korea	Event Time (UTC) yy-mm-dd Time	Latitude (°)	Longitude (°)	*M_L_*
1	Sangju	2020-01-29 15:52:52	36.59	128.12	3.20
2	Milyang	2019-12-29 15:32:08	35.56	128.90	3.45
3	Goesan	2022-10-28 23:27:49	36.88	127.88	4.12
4	Donghae	2019-04-19 02:16:43	37.88	129.54	4.27
5	Gyeongju	2016-09-19 11:33:58	35.74	129.18	4.50
6	Pohang	2018-02-10 20:03:03	36.08	129.33	4.60
7	Ulsan	2016-07-05 11:33:03	35.51	129.99	5.00
8	Gyeongju	2016-09-12 10:44:32	35.77	129.19	5.10
9	Pohang	2017-11-15 05:29:31	36.11	129.37	5.40

**Table 3:** Magnitude, Location, Occurrence Time Error between Real and Virtual Earthquakes by ElarmS (Earthquake early warning System) simulation experiments.

Case No.	Event Region in South Korea	Time Error (s)	Location Error (km)	Magnitude Error
1	Sangju	+0.11	1.64	−0.05
2	Milyang	+0.40	3.73	−0.07
3	Goesan	−0.04	1.64	−0.41
4	Donghae	−0.57	3.04	−0.02
5	Gyeongju	+0.51	3.14	+0.37
6	Pohang	+0.73	0.61	+0.44
7	Ulsan	−0.57	2.99	+0.67
8	Gyeongju	+0.75	2.23	+0.05
9	Pohang	+1.41	3.34	+0.56

## Data Availability

The earthquake record data can be downloaded from http://necis.kma.go.kr and https://www.kyoshin.bosai.go.jp, and the developed program will be provided upon request.
